# Effect of acupuncture at 3-points for intelligence on vascular dementia: Protocol for a systematic review and meta-analysis of randomized controlled trials

**DOI:** 10.1097/MD.0000000000012892

**Published:** 2018-10-19

**Authors:** Weipeng Sun, Minying Li, Tong Lin, Zhizhong Sun, Yizi Xie, Shuliang Ji, Jietao Lin, Lin Wang, Chao Jia, Liang Zheng, Wei Wu, Danghan Xu

**Affiliations:** a First Clinical College; b Medical College of Acu-Moxi and Rehabilitation, Guangzhou University of Chinese Medicine; c The First Affiliated Hospital of Guangzhou University of Chinese Medicine; d South China Research Center for Acupuncture and Moxibustion, Medical College of Acu-Moxi and Rehabilitation, Guangzhou University of Chinese Medicine, Guangzhou, China.

**Keywords:** acupuncture, meta-analysis, protocol, systematic review, vascular dementia

## Abstract

**Background:**

Vascular dementia (VD) is a commonly-seen disease in the elderly. What is more, “Acupuncture at 3-points for intelligence” is one of the most important components of “Jin's three-needle therapy” created by Rui Jin, a professor of Guangzhou University of Chinese Medicine, which can be used in the VD patients. In this article, researchers will assess the clinical efficacy and safety of acupuncture at 3-points for intelligence in the treatment of VD.

**Methods:**

A systematic literature search for articles up to September 2018 will be conducted using 9 databases: PubMed, Cochrane Library, Embase, CNKI, CBM, VIP, Wanfang database, OASIS, and CiNii. Inclusion criteria are randomized controlled trials (RCTs) of acupuncture at 3-points for intelligence on treating VD. The primary outcome measures will be scores reflecting the neurological function of participants based on common medical scales. Hemorheology indexes, homocysteine (Hcy), acetylcholine (Ach), nitric oxide (NO), and adverse events will also be assessed. Stata V.13.0 software will be used for data synthesis, sensitivity analysis, meta-regression, subgroup analysis, and risk of bias assessment. A funnel plot will be developed to evaluate reporting bias. Egger and Begg tests will be further performed to conduct quantitative evaluation of publication bias and to evaluate the symmetry of funnel plot. We will use the Grading of Recommendations Assessment, Development, and Evaluation system to assess the quality of evidence.

**Results:**

The results of this systematic review and meta-analysis will be published in a peer-reviewed journal.

**Conclusion:**

Our study will provide the evidence for the clinical efficacy and safety of acupuncture at 3-points for intelligence in the treatment of VD.

**Key Points**

- This study will assess the clinical efficacy and safety of acupuncture at 3-points for intelligence in the treatment of Vascular Dementia.
- Two reviewers will independently conduct the data extraction and risk of bias assessment.
- The Grading of Recommendations Assessment, Development, and Evaluation system will be applied to further evaluate study findings.
- There may be a language bias, as both English and Chinese studies will be included.
- There may be clinical heterogeneity due to variations in treatment frequency and duration and the use of additional therapies.

## 1 Introduction

### 1.1 Description of the condition

With a high incidence in the elderly, vascular dementia (VD) is characterized by chronic brain ischemia and progressive memory decline.^[1]^ Some findings have shown that obesity, underweight, diabetes, hyperlipidemia, smoking, atrial fibrillation, hyperhomocystinemia as well as hypertension are possible vascular risks for VD.^[2,3]^ Besides, the metabolic syndromes, including insulin resistance, hypertension, and dyslipidemia, are connected to lower cognitive performance which increases the odds of dementia onset.^[4]^ And one of the most important pathological factors of VD is poststroke, for the reason that transient ischemic attack does huge harm to cerebral vessels.^[5]^ VD not only shorten human life, but also lower patients and their family's quality of life, even impose economic burden.^[5,6]^ Current evidence represented that antihypertensives and statins might cut down the incidence of dementia.^[7]^ However, there are only few effective therapies for VD which the underlying mechanism of its pathology remains unclear.^[1]^ Fortunately, the ancient Chinese healing technique, acupuncture therapy—acupuncture at 3-points for intelligence, which is a kind of JIN's 3-needle therapy, are widely used in treating VD patients in China.^[8]^ What's more, acupuncture is generally considered safe when performed correctly.^[9]^

### 1.2 Description of the intervention

**Figure 1:**
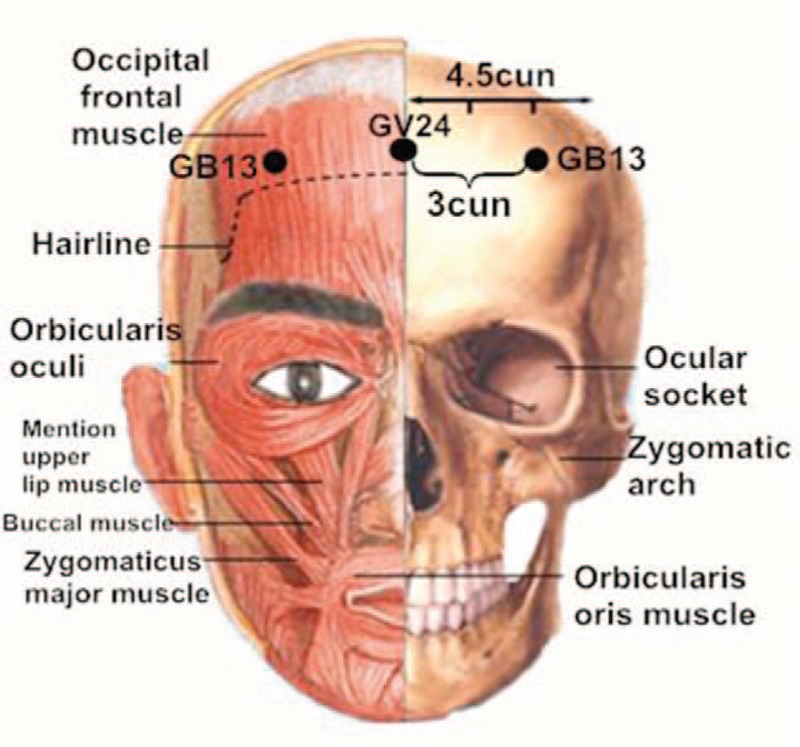
The anatomical location of GV24 and GB13.

**Figure 2:**
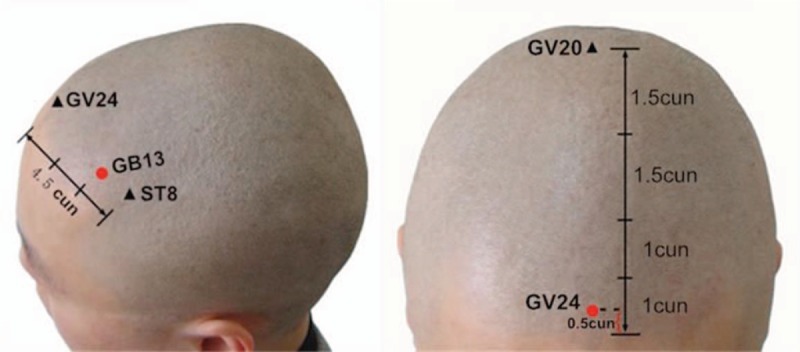
The location of GV24 and GB13.

**Figure 3:**
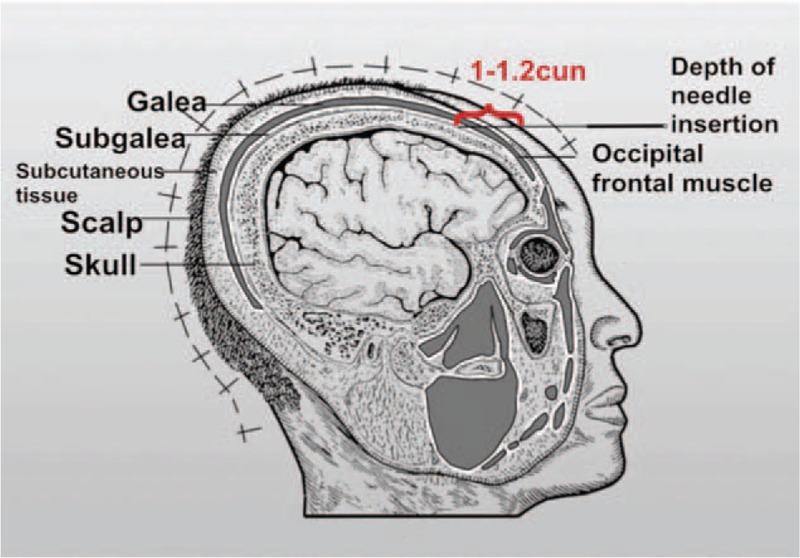
Depth of needle insertion of GV24 and GB13.

“Acupuncture at 3-points for intelligence” is one of the most important components of “Jin's 3-needle therapy” created by Rui Jin, a professor of Guangzhou University of Chinese Medicine. Acupuncture at 3-points for intelligence is composed of 3 acupoints: Shenting (GV24) and Benshen (GB13,bilateral).^[10]^ GV24 is located on the head, 0.5 cun directly above the midpoint of the anterior hairline. GB13 is located on the head, 0.5 cun above the anterior or hairline, 3 cun lateral to GV24, at the junction of medial two-thirds and lateral third of the line connecting GV24 and ST8, which is shown in Figs. 1 and 2. Cun is defined based on the rules of traditional acupuncture as the width of the interphalangeal joint of the patient's thumb.^[11]^ As it is shown in Fig. 3, needling is done subcutaneously from the front toward the back of the cranium parallel to the midsagittal plane to a depth of 1 to 1.2 cun to the subgalea for adult. The needle will be manipulated by lifting and twirling until the participant feels a sensation (denominated de-qi). Pay attention to avoid any visible subcutaneous cranial veins. When removing the needles one must apply pressure to the needle hold for a while so as to avoid subcutaneous bleeding. The quality of acupuncture depends on the patient's specific condition. Jin's 3-needle therapy has been used to cure children's mental retardation. It is beneficial in improving the children patients’ verbal comprehension, expression ability, hand-eye coordination ability, attention, logical reasoning ability, and visual perception.^[12]^ Furthermore, acupuncture at 3-points for intelligence has been proven to have a definite clinical effect on the treatment of primary insomnia^[13]^ and poststroke depression.^[14]^ Many clinical trials have shown that acupuncture treatment can improve the cognition by removing oxidized biochemical substances and promoting the release of neurotransmitters, which revealed that the mechanism of promoting angiogenesis in acupuncture and moxibustion is the core of VD treatment.^[15,16]^ There have been no relevant systematic reviews and meta-analyses on the effects of acupuncture at 3-points for intelligence on the treatment of VD. In addition, there is insufficient evidence to support the widespread application of acupuncture at 3-points for intelligence on the treatment of VD. Consequently, an exploration of this therapy's effect on VD is needed. In this study, researchers plan to conduct a systematic review and meta-analysis to evaluate current evidence on the effects of acupuncture at 3-points for intelligence on VD.

Researchers will include randomized controlled trials (RCTs) with no language restrictions that compare the effects of acupuncture or electroacupuncture with usual care or placebo acupuncture.

## 2 Methods and analysis

### 2.1 Eligibility criteria

#### 2.1.1 Study type

We will include all RCTs to assess the safety and efficacy of acupuncture at 3-points for intelligence therapy in the treatment of vascular dementia, regardless of the course of treatment, the age, course of disease, gender, and ethnicity of the patients. There is no restriction on language or publication status.

#### 2.1.2 Participants

We will select patients with both cerebrovascular disease and dementia, in which cerebrovascular disease is the prominent but not the only absolute cause of dementia. All patients should be diagnosed as VD according to at least one of the current or past definitions or guidelines of VD, such as:

1. Diagnostic and Statistical Manual of Mental Disorder 4th, DSM-IV.
2. National Institute of Neurological Disease and Stroke—Association Internationale pour la Rechercheer l’Enseignement et Neurosciences, NINDS-AIREN.
3. International Classification of Disease, ICD-10-R.
4. Alzheimer disease diagnosis and treatment center, ADDTC.

There will be no limitation on age, sex, and ethnicity of participants.

#### 2.1.3 Types of interventions

##### 2.1.3.1 Experimental interventions

We will include RCTs using only acupuncture at 3-points for intelligence (including ordinary acupuncture and electroacupuncture) or acupuncture at 3-points for intelligence with routine treatment of the control group. The difference between acupuncture at 3-points for intelligence and traditional acupuncture is that the traditional acupuncture involves inserting needles into classic acupoints of traditional meridian, while acupuncture at 3-points for intelligence only select 3 acupoints at a time, Shenting (GV24) and Benshen (GB13, bilateral). Some RCTs will be excluded (for example, fine needle, electrical stimulation, or hand). There is no restriction on the length and frequency of treatment.

##### 2.1.3.2 Comparator interventions

The control intervention will include sham acupuncture, placebo acupuncture, no treatment, medication (such as acetylcholinesterase inhibitors, excitatory amino acid antagonists, butylphthalide), or other active treatments (such as, lifestyle, dietary modification).

We will analyze the following comparisons:

1. Acupuncture at 3-points for intelligence monotherapy compared with sham or placebo treatment.
2. Acupuncture at 3-points for intelligence monotherapy compared with black control group.
3. Acupuncture at 3-points for intelligence plus another medication or active treatment compared with another medication or active treatment.
4. Acupuncture at 3-points for intelligence plus another medication or active treatment compared with sham or placebo treatment plus another medication or active treatment.

#### 2.1.4 Outcome measures

The primary outcome measurement will be scores reflecting the neurological function of participants based on certain common scales: minimental state examination (MMSE), Activity of Daily Living Scale (ADL), Montreal Cognitive Assessment (MoCA), Hachinski Ischemic Scale (HIS), Hastgawa Dementia Scale (HDS), Blessed dementia scale (BDS), National Institutes of Health Stroke Scale (NIHSS), Hamilton depression scale (HAMD) and so on.

The secondary outcome measurement will be hemorheology indexes, Hcy, Ach, NO, and adverse events.

### 2.2 Information sources

#### 2.2.1 Search strategy and identification of studies

We will retrieve literature in the following databases from their respective inception to September 2018: PubMed, Cochrane Library, Embase, 4 Chinese databases (CNKI, CBM, VIP, and Wanfang database), 1 Korean medical database (OASIS), and 1 Japanese medical database (CiNii). The search terms will be “vascular dementia” OR “vascular cognitive impairment” OR “VD” AND “Zhi 3-needle” OR “Zhisan Needle” OR “Zhisanzhen” OR “Jin's 3-needle” OR “Jin's 3-needle” OR “3-points for intelligence” OR “acupuncture” OR “moxibustion” OR “needle” OR “electroacupuncture.” The Chinese, Japanese and Korean translations of these terms will be used for databases search of corresponding countries. The search strategy for PubMed is given in Table 1.

**Table 1:**
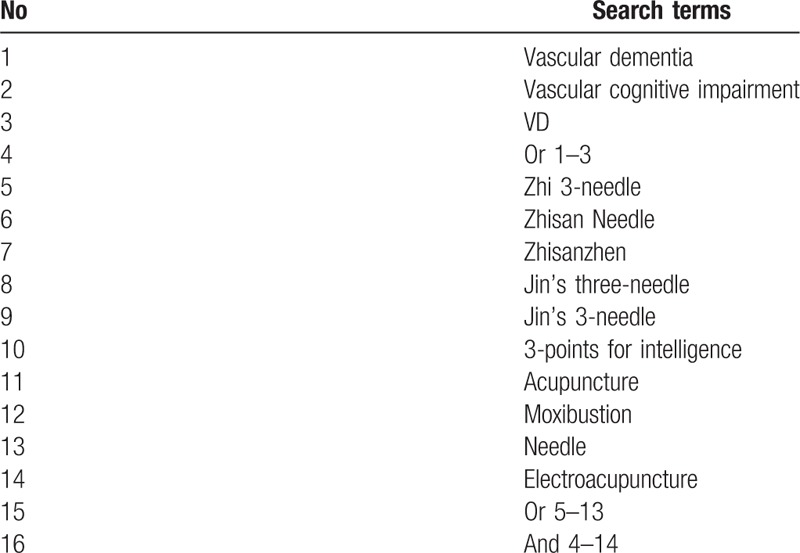
Search strategy utilized for the PubMed database.

**Figure 4:**
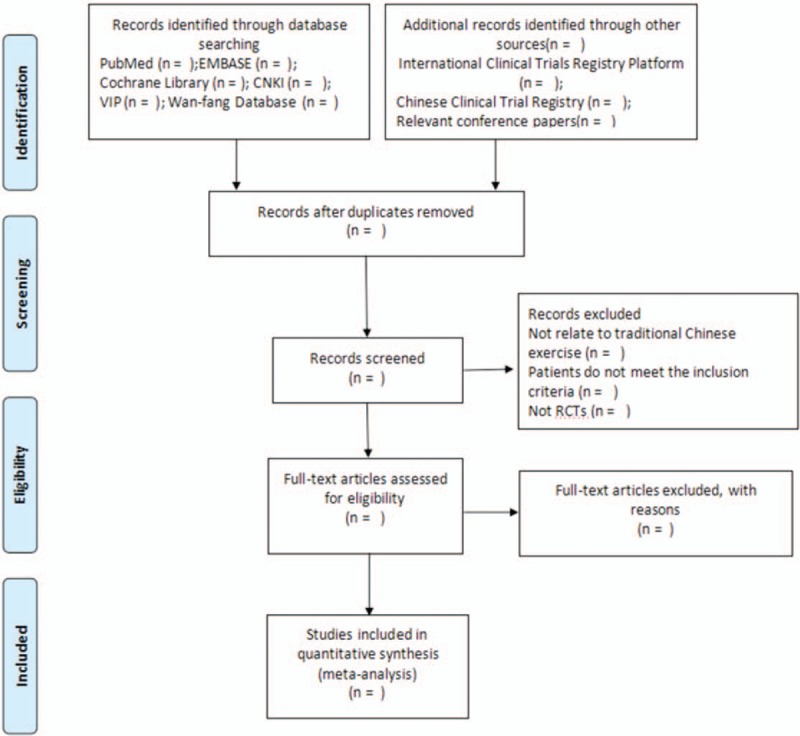
Flow diagram of the study selection process.

Two reviewers (TL and ZS) will independently scan the full texts of potential eligible studies. A third reviewer will discuss and settle the discrepancies about inclusion in the meta-analysis. And we will produce Preferred Reporting Items for Systematic Reviews and Meta-Analyses (PRISMA) flow chart to set out the number of articles identified, screened, included, and excluded. Reasons for exclusion and ascertaining eligible studies will also be shown. The study selection process will completely be stated in a PRISMA flow chart (http://www.prisma-statement.org) (Fig. 4).

#### 2.2.2 Study selection

We will exclude articles of which no data on scales outcomes are presented, results are reused, or related information are not available. Two reviewers (TL and ZS) will independently screen titles and abstracts to select potential references according to the criteria mentioned above. If necessary, reviewers will examine full texts to identify eligible studies. Endnote V.X7 software will be utilized to manage literature and remove duplicate literature.

### 2.3 Data extraction

The reviewers will extract the characteristics of the included studies (about the subjects, interventions, results) by using a self-developed data extraction table. The following items will be included in the data extraction form: title, first author, funding year of publication, country of study, design, inclusion and exclusion criteria, methods of randomization, allocation concealment, age, sex, sample size, dose, follow-up time points, compliance, primary and secondary outcomes, method of outcome assessments, blinding of outcome assessment, adverse events. Any disagreement will be resolved by consensus or consultation with the third reviewer.

#### 2.3.1 Addressing missing data or unclear measurement scales

If necessary, we will attempt to contact authors of studies by email or telephone for missing data or clarification about evaluation scales. If we could not obtain sufficient information in this way, we will analyze the available data. We will try to evaluate the potential influence of missing data on the review results (in the Discussion section).

### 2.4 Risk of bias in included studies

Risk of bias for each included RCTs will be assessed by 2 authors (ML and SJ) according to the Cochrane Handbook for Systematic Reviews of Interventions. This recommends the assessment of several sources of bias, including selection bias, performance bias, detection bias, attrition bias, reporting bias, and other bias. We will try to describe the risk of bias reported in each study. Thus, we will be able to judge the risks as low, high, or unclear (unclear or unknown risk of bias). If necessary, we will contact authors of included articles for further information.

### 2.5 Statistical analysis

#### 2.5.1 Data synthesis and analysis

Stata V.13.0 software will be applied for data analysis. 95% CIs and weighted mean differences will be calculated. Risk ratio with 95% CIs will be used to express the estimate of the effect for dichotomous outcomes. For continuous outcomes, we will express the estimate of the effect as mean difference with 95% CIs. The Q and I^2^ test statistics will be performed to evaluate the heterogeneity of the included studies. For the Q statistic, *P* < .05 will be considered as indicating significant differences. For the I^2^ statistic, I^2^ < 25% indicates no significant heterogeneity, I^2^ = 25% to 50% is considered moderate heterogeneity and I^2^ > 50% indicates strong heterogeneity. If there is heterogeneity, data will be analyzed with the random effects models, otherwise, the fixed effects models will be adopted.

#### 2.5.2 Additional analyses

We will explore the possible sources of heterogeneity by conducting sensitivity analysis, partial sequence analysis, and subgroup stratification analysis. The process needs to be implemented according to the different research features, such as research types, locations, racial difference, sample size, gender differences, quality of research, adjustable or unadjustable confounding factors, treatment, and other related parameters. If the extracted data is insufficient for quantitative analysis, qualitative analysis will be carried out.

#### 2.5.3 Assessment of reporting biases

A funnel plot will be developed using software to qualitatively assess the reporting bias of the included studies. We will further use Egger and Begg tests to conduct quantitative evaluation of publication bias and to evaluate the symmetry of funnel plot. We interpret the value of *P* < .1 as having statistical significance (i.e., publication bias).

### 2.6 Quality of evidence

The quality of the evidence of the included studies will be evaluated according to the Grading of Recommendations Assessment, Development, and Evaluation (GRADE) approach. Due to the possible limitations of the study, inconsistencies, inaccuracies, indirect evidence and reporting bias, the quality of evidence for each study will be graded as 4 levels: high, moderate, low, or very low.

## 3 Ethics and dissemination

Our expected goal is to publish this systematic review and meta-analysis in a peer-reviewed journal. Our article will provide information on the safety and efficacy of acupuncture at 3-points for intelligence in treating VD patients compared with other conventional treatments. This study will not involve participants’ privacy, therefore ethical approval is not required.

## 4 Discussion

Acupuncture at 3-points for intelligence is a form of acupuncture that has been used in clinical practice for more than 30 years in China.^[17]^ Acupuncture at 3-points for intelligence is effective and safe which is suitable for patients with VD.^[18,19]^ In particular, the effect of electroacupuncture at 3-points for intelligence on VD is extremely significant.^[20]^ Previous studies have indicated that acupuncture at 3-points for intelligence can obviously improve the intelligence level and cognitive function of patients with VD. The incubation stage of cognitive evoked potential P_300_ and the amplitude of N_2_ and –P_8b_ are significantly improved, indicating that the cognitive functions of patients are significantly ameliorated.^[16]^ One study has reported that acupuncture is considered a preferable alternative pharmacotherapy for treating relevant outcomes in dementia such as behavioral disturbances.^[21]^ Research has also reflected a beneficial effect of additional acupuncture on cognitive status and activities of daily living for VD patients.^[22]^

Acupuncture at 3-points for intelligence has been developed to treat VD, but the efficacy of the therapy compared with medications for VD is unknown. From a medical perspective, treatments, and/or management of dementia and its prevention require a long-term care rather than short-term intensive treatment. Therefore, compliance, economic costs, safety, and efficacy of the interventions are quite important. The purpose of this review is to systematically assess the effect of acupuncture at 3-points for intelligence on treating VD. We aim to collect enough studies to ensure the meta-analysis more convincing. We expect to find that acupuncture at 3-points for intelligence therapy has a positive effect on the treatment of VD. In summary, this review will be the first to evaluate the impact of acupuncture at 3-points for intelligence on the treatment of VD. The results of this review may help to establish a better approach to treat VD and provide reliable evidence for its extensive application.

## Author contributions

WS, ML, and DX conceived the study and drafted the protocol. JL, CJ, LZ, and WW revised it. TL, ZS, YX, SJ, and LW developed the search strategies, conducted data collection, and analyzed independently. All authors have approved the final manuscript.

**Conceptualization:** Tong Lin, Danghan Xu.

**Formal analysis:** Zhizhong Sun, Liang Zheng.

**Investigation:** Shuliang Ji.

**Methodology:** Yizi Xie, Lin Wang.

**Resources:** Tong Lin.

**Software:** Jietao Lin.

**Writing – original draft:** Weipeng Sun, Minying Li.

**Writing – review & editing:** Chao Jia, Wei Wu.

## References

[1] O’Brien JT Thomas A Vascular dementia. Lancet 2015;386:1698–706. 26595643 10.1016/S0140-6736(15)00463-8

[2] Hasnain M Vieweg WV Possible role of vascular risk factors in Alzheimer's disease and vascular dementia. Curr Pharm Des 2014;20:6007–13. 24641219 10.2174/1381612820666140314153440

[3] Sahathevan R Brodtmann A Donnan GA Dementia, stroke, and vascular risk factors; a review. Int J Stroke 2012;7:61–73. 22188853 10.1111/j.1747-4949.2011.00731.x

[4] Yates KF Sweat V Yau PL Impact of metabolic syndrome on cognition and brain: a selected review of the literature. Arterioscler Thromb Vasc Biol 2012;32:2060–7. 22895667 10.1161/ATVBAHA.112.252759 PMC3442257

[5] Kalaria RN Akinyemi R Ihara M Stroke injury, cognitive impairment and vascular dementia. Biochim Biophys Acta 2016;1862:915–25. 26806700 10.1016/j.bbadis.2016.01.015 PMC4827373

[6] Van De Vorst IE Vaartjes I Geerlings MI Prognosis of patients with dementia: results from a prospective nationwide registry linkage study in the Netherlands. BMJ Open 2015;5:e008897. 10.1136/bmjopen-2015-008897 PMC4636675 26510729

[7] Barca ML Engedal K Laks J Quality of life among elderly patients with dementia in institutions. Dement Geriatr Cogn Disord 2011;31:435–42. 21757909 10.1159/000328969

[8] Larsson SC Markus HS Does treating vascular risk factors prevent dementia and Alzheimer's Disease? A systematic review and meta-analysis. J Alzheimers Dis 2018;64:657–68. 29914039 10.3233/JAD-180288

[9] Li J Zhang X Han J Treatment for vascular dementia by acupuncture and moxibustion. J Clin Acupuncture Moxibustion 2012;3:72–4.

[10] Chan M Wu X Wu J Safety of acupuncture: overview of systematic reviews. Sci Rep 2017;7:3369. 28611366 10.1038/s41598-017-03272-0 PMC5469776

[11] Melchart D Streng A Hoppe A Acupuncture in patients with tension-type headache: randomized controlled trial. BMJ 2005;331:376–82. 16055451 10.1136/bmj.38512.405440.8F PMC1184247

[12] Zeng X Li Q Xu Q Acupuncture mechanism and redox equilibrium. Evid Based Complement Alternat Med 2014;2014:483294. 25097658 10.1155/2014/483294 PMC4109597

[13] Huang X Yuan Q Luo Q Clinical efficacy on mental retardation in the children treated with JIN's three scalp needling therapy and the training for cognitive and perceptual disturbance. Zhongguo Zhen Jiu 2015;35:651–6. 26521572

[14] Yang H Huang F Kuang W Efficacy of acupuncture at Zhisanzhen in treatment of primary insomnia: a report of 35 cases. J Anhui Univ Chin Med 2014;3:74–6.

[15] Tian X Zhang Q Xu S A study of clinical efficacy evaluation of Zhisanzhen as primary acupuncture in treating patients with post-stroke depression. Chin J Integr Traditional Western Med Intensive Crit Care 2011;4:219–21.

[16] Xie F Xu Z Xiong X Effect of mental tri-needle electro-acupuncture on postoperative cognitive function in elderly patients after hip joint replacement surgery. J Guangzhou Univ Traditional Chin Med 2016;6:813–7.

[17] Li Q Li L Xu Q Effect of electroacupuncture at Zhisanzhen on cognitive function and ability of life activity in patients with vascular dementia. Chinese J Pract Nervous Dis 2015;8:1–2.

[18] Jin R Lai X Wang S Clinical observation on 558 cases of mentally retarded children treated with Sishen needle and Zhi three-needle. Zhong Guo Zhen Jiu 1992;2:3–5.

[19] Zhou F Zeng K Clinical study of Zhi three-needle combined with hyperbaric oxygen in the treatment of vascular dementia. Hunan J Traditional Chin Med 2015;8:82–3.

[20] Tang Z Lu Y Song H Effects of electroacupuncture at Zhi three- needle on learning and memory ability and β-Amyloid expression in VD rats. Chinese J Gerontol 2012;13:2794–6.

[21] Abraha I Rimland JM Trotta FM Systematic review of systematic reviews of non-pharmacological interventions to treat behavioural disturbances in older patients with dementia. BMJ Open 2017;7:e012759. 10.1136/bmjopen-2016-012759 PMC5372076 28302633

[22] Shi G Li Q Yang B Acupuncture for vascular dementia: a pragmatic randomized clinical trial. Scientific World J 2015;2015:161439. 10.1155/2015/161439 PMC4606072 26495416

